# Quantitative comparison of a mobile and a stationary video-based eye-tracker

**DOI:** 10.3758/s13428-019-01267-5

**Published:** 2019-06-25

**Authors:** Stefan Dowiasch, Peter Wolf, Frank Bremmer

**Affiliations:** 1 Department of Neurophysics, Philipps-Universität Marburg, Marburg, Germany; 2 Center for Mind, Brain and Behavior (CMBB), Philipps-Universität Marburg, Marburg, Germany; 3 Thomas RECORDING GmbH, Gießen, Germany

**Keywords:** Eye-tracking, Quantitative comparative analysis, Saccade eye movements, Smooth-pursuit eye movement (SPEM), Fixation eye movement, Mobile eye-tracking, Accuracy evaluation methods, Eye movement analysis

## Abstract

Vision represents the most important sense of primates. To understand visual processing, various different methods are employed—for example, electrophysiology, psychophysics, or eye-tracking. For the latter method, researchers have recently begun to step outside the artificial environments of laboratory setups toward the more natural conditions we usually face in the real world. To get a better understanding of the advantages and limitations of modern mobile eye-trackers, we quantitatively compared one of the most advanced mobile eye-trackers available, the EyeSeeCam, with a commonly used laboratory eye-tracker, the EyeLink II, serving as a gold standard. We aimed to investigate whether or not fully mobile eye-trackers are capable of providing data that would be adequate for direct comparisons with data recorded by stationary eye-trackers. Therefore, we recorded three different, commonly used eye movements—fixations, saccades, and smooth-pursuit eye movements—with both eye-trackers, in successive standardized paradigms in a laboratory setting with eight human subjects. Despite major technical differences between the devices, most eye movement parameters were not statistically different between the two systems. Differences could only be found in overall gaze accuracy and for time-critical parameters such as saccade duration, for which a higher sample frequency is especially useful. Although the stationary EyeLink II system proved to be superior, especially on a single-subject or even a single-trial basis, the ESC showed similar performance for the averaged parameters across both trials and subjects. We concluded that modern mobile eye-trackers are well-suited to providing reliable oculomotor data at the required spatial and temporal resolutions.

The human brain is continuously exposed to external sensory stimuli and has to process them in order to interact with our environment. The main sensory input of primates is the visual sense (Palmer, 1999), which uses the eyes to gather visual information from the environment. One functional property of the eyes that stands out among the other sensory organs is the ability to move. We constantly make eye movements in order to analyze certain aspects of our visual field with the part of the retina with the highest possible resolution, the fovea. About three times per second (Land, Mennie, & Rusted, 1999; Rayner, 1998)—that is, more often than our heart beats—we change our gaze, with a fast, ballistic eye movement called a *saccade* (Carpenter, 1988).

For more than 100 years, eye movements and their dysfunctions were utilized by researchers in order to investigate general brain functions (Helmholtz, 1866; Dodge, 1903; Yarbus, 1967) and specific impairments in such diseases as schizophrenia or Parkinson’s disease (Diefendorf & Dodge, 1908; Jones & DeJong, 1971; Leigh & Zee, 2006). Ever since, eye movements have been proven to serve as a “window on mind and brain” (Van Gompel, Fischer, Murray, & Hill, 2007). The noninvasive, reliable, rapid, and simple measurement of eye movements offers the unique opportunity to gain deeper insights into the underlying mechanisms of brain function (Coe & Munoz, 2017; Ilg, 1997; Krauzlis, 2004; Leigh & Kennard, 2004). A great part of our understanding of the visual system originated from animal models, especially nonhuman primates. Due to the monkey’s considerable similarity to humans with regard to visual processing, it is possible to extrapolate results from monkey to men (Bremmer et al., 2001; Felleman & Van Essen, 1991; Fuchs, 1967; Orban, Van Essen, & Vanduffel, 2004; Solomon & Rosa, 2014). These functional similarities allow us to reliably identify neuronal correlates of specific aspects of vision, from the neuronal basis of eye movements (Krauzlis, 2004; Thier & Ilg, 2005), to more cognitive processes such as attention (Treue, 2001). On the basis of this previous work, it is possible to trace specific areas in the brain using eye movement abnormalities, which can be observed in many different neurological diseases, or can be elicited artificially in psychophysical experiments with healthy participants by pushing the visual system to its limit (Gescheider, 2013). As a result, the sole measurement of eye movements is capable of identifying possible sources of neuropsychiatric diseases as well as extending the knowledge of specific aspects of vision in the healthy brain (e.g., Dowiasch, Backasch, et al., 2016a; Dowiasch, Marx, Einhäuser, & Bremmer, 2015). In the long run, eye movements might even provide objective parameters to support the diagnosis of specific brain diseases in the clinical routine (Benson et al., 2012; Marx et al., 2012; Tseng et al., 2013).

Eye movement studies are typically performed in the laboratory with artificial visual stimuli. Laboratory settings, however, might have an influence on the oculomotor performance of the participants, especially of neuropsychiatric patients (Dowiasch, Backasch, et al., 2016a). Hence, there is an urgent need to monitor eye movements at high spatial and temporal resolution outside the laboratory. Indeed, with the growing availability of mobile eye-trackers in recent years, scientists began to investigate eye movements in more realistic settings (Hayhoe, 2000; Land Land, Mennie, & Rusted, 1999). Importantly, among others, this approach, allows to determine to what extent common findings from the laboratory can be transferred to activities of daily living.

Recent studies on eye movements comparing results from the laboratory with those from real-world scenarios found significant differences between each other (Foulsham, Walker, & Kingstone, 2011; Marius't Hart et al., 2009; Tatler, Hayhoe, Land, & Ballard, 2011). Moreover, eye movements in the real world have been shown to be generally more variable across subjects. This might be due to the fact that tasks in the real world are usually much less constrained than tasks in the laboratory, to allow subjects to behave more naturally. However, this freedom of performing a general task—for example, making a cup of tea—can result in quite different eye movement behavior, which can be modulated even further by different tasks (Hayhoe, 2000; Land, Mennie, & Rusted, 1999). As an example, when there is no specific task, eye movements are mainly performed along the two main axes within the visual field (horizontal and vertical; Einhäuser et al., 2007). Furthermore, natural environments are much harder to control than laboratory setups in which a task can be controlled to almost every extent, from a specific timing to minimization of confounds. This can have a different impact on subjects, on how they perform the tasks, on how they react to distractions and therefore on their specific eye movement patterns. For instance, real world eye movements are highly modulated by different environments (Pelz & Rothkopf, 2007; Marius't Hart & Einhäuser, 2012). These and other findings raised a question concerning a general transferability of results from eye movement measurements in the laboratory to the real world. Simultaneously they underline the need to better understand oculomotor behavior in natural environments. Additionally, in contrast to the restrained conditions in the laboratory, eye movements in more natural settings are typically accompanied by head movements (Einhäuser et al., 2009; Land & Fernald, 1992). As such, eye movements often compensate for head movements to stabilize gaze or aid head movements in performing huge gaze shifts (Goossens & Van Opstal, 1997; Guitton, 1992).

Furthermore, participants in laboratory setups are usually artificially deprived of additional sensory input other than the aspect under investigation, whereas information processing of the visual system in the real world has to cope with additional sensory signals providing—for example, vestibular, auditory, or tactile information. These differences might contribute to the different eye movement behaviors found in the laboratory and in more natural settings (Dowiasch, Backasch, et al., 2016a). Accordingly, it is of critical importance to correctly attribute potential differences in eye movement behavior to the environmental conditions.

Finally, in addition to all these behavioral differences found in eye movement studies in natural environments and laboratory settings, it is of the utmost importance to evaluate any technical differences between mobile and laboratory-based eye-trackers—for example, in terms of accuracy and the typical procedure to analyze the data. Only if one can guarantee that both systems are able to generate data of similar quality, and that these data are analyzed with similar algorithms, can results from laboratory settings be directly compared to those from natural environments without further restriction. In this study we quantitatively compared one of the most sophisticated mobile eye-trackers available, the EyeSeeCam (ESC; Schneider et al., 2009), with a commonly used stationary but head-mounted laboratory eye-tracker, the EyeLink II (ELII), serving as gold standard in oculomotor research. We aimed to determine whether fully mobile eye-trackers are capable of providing adequate data with accuracy comparable to established laboratory-based eye-trackers in prototypical eye movement paradigms to allow for a direct comparison with data from stationary video-based eye-trackers. Furthermore, we compared the default analysis procedures of the two eye-tracking systems in order to identify potential differences arising from different preprocessing of the raw data.

## Method

For both eye-trackers, the ESC and the ELII, we investigated the same basic eye movement parameters during three different types of eye movements, commonly utilized in oculomotor studies—that is, steady fixation, saccades, and smooth-pursuit eye movements. Eight subjects, four males and four females, with a mean age of 21.3 ± 1.8 years, participated in the study. This study was carried out in accordance with the recommendations of the ethics committee of the department of psychology (reference number: 2012-23K) with written informed consent from all subjects. All subjects gave written informed consent in accordance with the Declaration of Helsinki. The protocol was approved by the ethics committee of the department of psychology of Philipps-Universität Marburg. Each subject had to perform the same standardized sequence of paradigms twice, each time with a different eye-tracker. Half of the subjects used the ESC first, the other half were first measured with the ELII. All participants had normal or corrected-to-normal vision and gave their written consent prior to the experiment. All measurements were performed in a dark and soundproofed room. The stimuli were projected by a video-projector (Christie DS + 6k-*M*) running at 120 Hz on a tangent screen (width: 1.20 m, height: 0.90 m, corresponding to 81° × 65° of visual angle), 70 cm in front of the subjects. Head movements were restricted by a chin rest throughout the entire measurement with both types of eye-trackers.

### Devices

Today numerous eye-tracking devices are available from different companies, both for mobile and for stationary eye-tracking. In this study we tried to identify one representative candidate for each field of application that is both state-of-the-art, relative to other devices within the field, and as similar as possible to each other in order to increase comparability. Table 1 shows four different commonly used, laboratory-based eye-trackers with their technical specifications according to the respective manuals. Table 2, on the other hand, lists four different mobile eye-tracking devices with their respective technical specifications. Since the environment in which the latter devices are used can have a significant influence on their performance, we also provided values on accuracy and precision from experiments in an indoor natural environment at three different viewing distances (MacInnes, Iqbal, Pearson, & Johnson, 2018), in addition to the parameters given by the manufactures.

**Table 1:** Technical specifications of four well-established and commonly used laboratory-based eye-trackers according to their respective manuals

	EyeLink II^a^	EyeLink Portable Duo^b^	SMI iView^c^	Tobii Pro Spectrum^d^
Gaze accuracy	0.5°	0.25°–0.5°	~ 0.3°	~ 0.3°
Gaze precision	0.01°	0.01°	0.01°	0.06°
Calibration procedure	3-, 5-, 9-, 13-point	3-, 5-, 9-, 13-point	1-, 5-, 9-, 13-point	1–14 points
Sampling frequency	500 Hz	2000 Hz	2000 Hz	1200 Hz
Signal latency	3 ms	1.88 ± 0.36 ms	n.a.	2.5 ms

*Accuracy* describes the difference between the measured and the true value, whereas *precision* describes the statistical variability in the measured data. The *calibration procedure* indicates how many targets can be used throughout the calibration process. *Sampling frequency* describes the maximum available temporal resolution of the eye-tracker and signal latency the time between an event and when this event is available as an output signal by the respective software. ^a^https://www.sr-research.com/products/eyelink-ii/. ^b^https://www.sr-research.com/products/eyelink-portable-duo/. ^c^Discontinued. ^d^https://www.tobiipro.com/product-listing/tobii-pro-spectrum/

**Table 2:** Technical specifications of four commonly used mobile eye-trackers according to their respective manuals or, marked in parentheses, results of recent comparative studies using them in natural indoor environments (MacInnes et al., 2018)

Eye-Tracker	EyeSeeCam^e^	Pupil labs^f^	SMI Eye-Tracking Glasses^g^	Tobii Pro Glasses 2^h^
Gaze accuracy (center)	0.5° (1.3°)	0.6° (0.8°)	0.5° (1.2°)	0.62° (1.4°)
Gaze precision	0.05° (0.5°)	0.08° (0.16°)	0.1° (0.19°)	0.05° (0.34°)
Calibration procedure	0-, 1-, 5-point	5- & 9-point	0-, 1-, 3-point	1-point
Sampling frequency	230 Hz	200 Hz	120 Hz	100 Hz
Signal latency	10 ms	7.5 ms	n.a.	> 10 ms

Gaze accuracy is only reported for a region ± 10° off from straight ahead in the vertical and horizontal direction, since some manufactures only provide these values. All other parameters are like those in Table 1. ^e^https://www.sr-research.com/products/eyelink-ii/;http://www.eyeseetec.de/eyeseecam-sci/. ^f^https://pupil-labs.com/pupil/. ^g^Discontinued. ^h^https://www.tobiipro.com/de/produkte/tobii-pro-glasses-2/

On the basis of these data, we chose the ELII system as the laboratory-based eye-tracker to serve as reference, since it is a well-established device in the field with sufficient accuracy and temporal resolution for most eye-tracking studies. More importantly, it is a head-mounted device just like most mobile eye-trackers. This might reduce differences in the results due to two completely different measurement approaches. For the mobile eye-tracking devices, we chose the ESC as a representative candidate. The ESC is a fully mobile, lightweight eye-tracker, which is able to record binocular eye movements with a sampling rate of 230 Hz, a spatial resolution of 0.02°, and a precision of up to 0.1° (Schneider et al., 2009). The ESC has been used recently in a number of studies—for example, to identify different eye movement behavior in neurological and psychiatric patients (Dowiasch, Backasch, et al., 2016a; Korsager, Schmidt, Faber, & Wanscher, 2016; Marx et al., 2012), or gaze patterns in everyday tasks (Dowiasch et al., 2015; Kugler et al., 2015; Stoll, 2015Marius't Hart & Einhäuser, 2012, 2012). The system was calibrated before each measurement using five predefined targets (one in each cardinal direction and one straight ahead), projected to a plain surface with a head-fixed laser pointer using a diffraction grating. This grating is constructed such that each peripheral target always has a vertical or horizontal distance of 7.5° from straight ahead, no matter what the distance between subject and projection surface is. The gaze direction of each subject was matched to the respective targets and validated afterward. The mean error threshold for a successful calibration was 0.5° for the central fixation point. The ELII system was operated binocularly with 500 Hz and calibrated with a nine-point raster, which was matched to the current gaze directions. The mean error threshold during the calibration was also set to 0.5°. However, there are large differences between the calibration procedures of the two eye-trackers that go beyond difference in the calibration targets used (see Tables 1 and 2). While laboratory-based eye-trackers like the ELII usually use a vector between the center of the dark pupil and the corneal reflection of the infrared illumination unit that gets mapped to the screen coordinates of the calibration targets, mobile eye-trackers like the ESC more often employ 3-D models of the eye ball. For the latter, the calibration procedure is used to adjust the parameters of this model. The former usually is more accurate, but requires some boundary conditions. As one example, there can be only one light source at a fixed position relative to the eye. Yet the 3-D model of the eye allows for measuring relative eye movement parameters like saccade amplitude even without any calibration with reasonable accuracy. This feature is especially useful for eye movement studies with patient groups, whose compliance is limited (Marx et al., 2012). Finally, it was not feasible to link the ESC to a stimulus computer to allow, for example, for online drift correction such as the ELII provides. Thus, the underlying procedures to compute, and, if applicable, update, eye positions differ significantly between typical laboratory-based and mobile eye-trackers. This might contribute to potential differences in the results obtained from the same paradigms with different eye-trackers. Especially the accuracy might be affected by this difference, since it heavily relies on a good calibration and is prone to shifts or slippage throughout the measurement. This has to be considered, and detailed functional properties of the devices have to be investigated, if one wants to compare or transfer results from the laboratory to those in the real world. In this study we provide a first insight into this issue.

### Paradigm

**Fig. 1:**
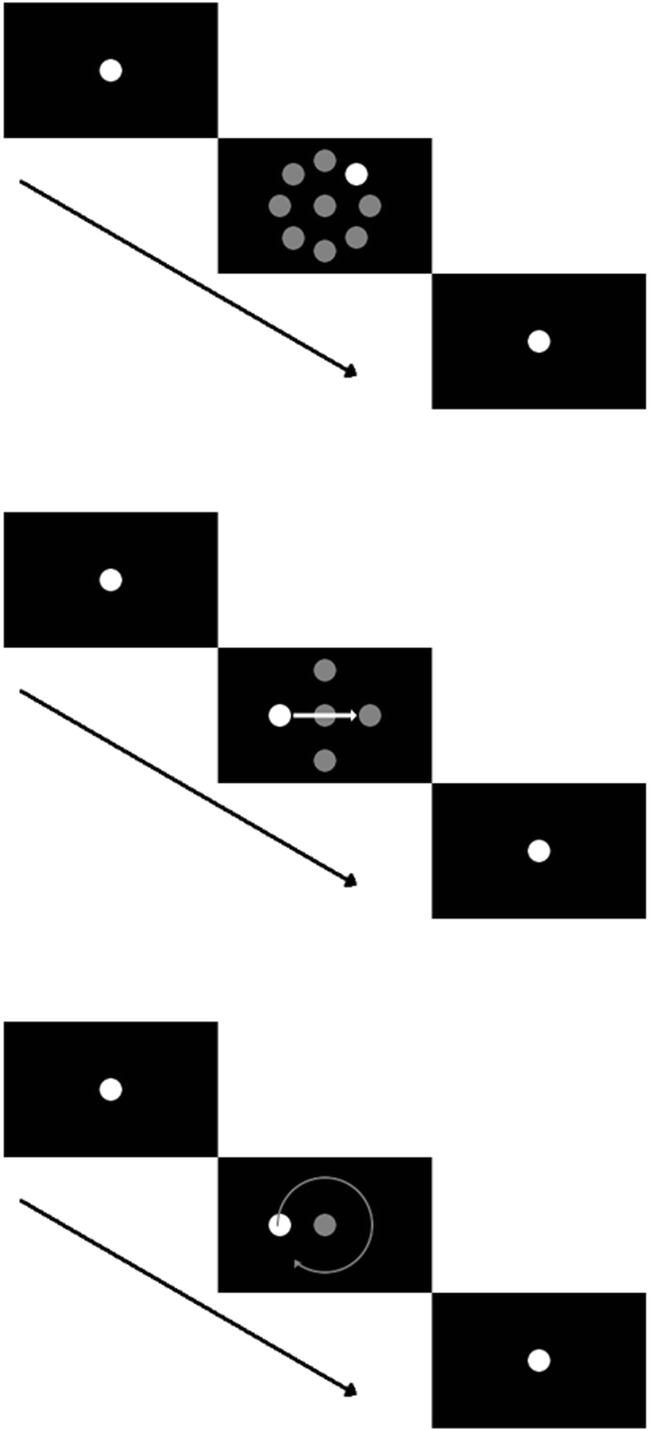
Schematic overview of the three different eye movement paradigms used. All paradigms started and ended with a fixation target presented for 1 s at the center of the screen. (A) *Saccade task:* The fixation target randomly jumped to one out of eight positions with an eccentricity of 10°, requesting subjects to perform a visually guided saccade. This eccentric target was displayed for 2 s, after which the target jumped back to the center of the screen. (B) *Step-ramp pursuit task:* The subjects had to perform a step-ramp pursuit in one of four directions (up, down, left, or right) with a velocity of 10°/s. To this end, the central fixation target jumped randomly to one out of four positions with an eccentricity of 10° and immediately started moving back toward the center. After 2 s, the pursuit target reached the position opposite from the initial target jump, from which it immediately jumped back toward the center of the screen. (C) *Circular-pursuit task:* The fixation target jumped to a random position on an imaginary circle with a radius of 5° and immediately started moving on this circle in a clockwise direction with an absolute velocity of 10°/s. After 5/4 orbits on this circle, corresponding to about 3.9 s, the target jumped back to the center of the screen

The experiment consisted of a saccade task including fixation periods (Fig. 1A) and two pursuit tasks (Figs. 1B and C), presented in separate sessions. All stimuli consisted of a white dot (luminance: 69.5 cd/m^2^, diameter: 0.5°) on a black background, which subjects had to fixate or track with their eyes when it moved. In the saccade task, subjects had to fixate a central dot for 1 s. Afterward, the target jumped into the periphery to one of eight predefined locations, 45° apart from each other on an imaginary circle with a radius of 10° (Fig. 1A). After 2 s, the target jumped back to the central position and remained there for another second. To investigate smooth-pursuit eye movements, two paradigms were used. First, a classical step-ramp paradigm (Rashbass, 1961) was employed, to elicit linear smooth pursuit. Here subjects fixated a central target for 1 s, after which it stepped toward one of four possible positions (up, down, left, right) 10° in the periphery and immediately began to move with a constant speed of 10°/s centripetally (Fig. 1B). After 2 s the pursuit target disappeared, and a central fixation point was shown for 1 s. The second pursuit paradigm elicited circular pursuit. Here, after an initial central fixation of 1 s, the target stepped into a random position in the periphery, on an imaginary circle with a radius of 5°, and immediately started to move clockwise along that circle with a speed of 10°/s (Fig. 1C). After 3.9 s, corresponding to 5/4 orbits, the pursuit target jumped back to the center of the screen and stayed there for 1 s. After each trial in each paradigm, the subject had to fixate the central fixation target and press the space bar in order to start the next trial. When measuring with the ELII system, this procedure was used as an online drift correction (SR Research Ltd, 2006).

To synchronize the ESC recording laptop with the stimulus PC, we presented a short trigger stimulus before each trial. This trigger stimulus was a 5°-wide gray square shown for four frames (33 ms). This square was detected offline in the video of the head-fixed scene camera of the ESC and served as a reference for the stimulus start in the internal timeline of the ESC. Only if the trigger stimulus was correctly detected in both datasets—that is, by an event in the data files of the ELII and in the video of the ESC—was the trial further analyzed.

### Data analysis

Eye movement parameters were analyzed offline using MATLAB 2015a. The preprocessing of the eye-movement data (e.g., saccade detection, filtering, etc.) typically differs between different eye-tracking systems and depends on their field of application. Therefore, in a first step, we used the standard data analysis procedures provided by the manufacturer (ELII) or established in the literature (ESC; Dowiasch, Backasch, et al., 2016a; Dowiasch et al., 2015; Marx et al., 2012). Additionally, in order to exclude blink artifacts, all data points from 40 ms before until 60 ms after a detected blink were excluded from further analysis in both systems.

***Saccades*** The ELII has a build-in online saccade and blink detector, which automatically marks those events in the recorded data. This detector uses a velocity criterion for saccades with a threshold of 30°/s and an additional acceleration threshold of 8,000°/s (SR Research Ltd, 2006). On the other hand, since the ESC is typically used in natural environments with unrestrained head movements, a much more robust saccade detector is needed. To exclude other eye movements like VOR or artifacts resulting from light reflexes from external sources, a higher velocity threshold for saccade classification is typically used (Dowiasch, Backasch, et al., 2016a; Dowiasch et al., 2015; Marx et al., 2012). Here the eye velocity had to exceed 100°/s for at least three successive data points (10.7 ms). This more conservative definition of a saccade might lead to fewer data points being attributed to a saccade, as compared to the ELII detector, resulting in slightly different saccade parameters, such as duration or amplitude. Furthermore, small corrective saccades—for example, catch-up saccades during pursuit eye movements—might be completely omitted by the ESC saccade detector, which was adapted to more uncontrollable real-world eye movements.

**Fig. 2:**
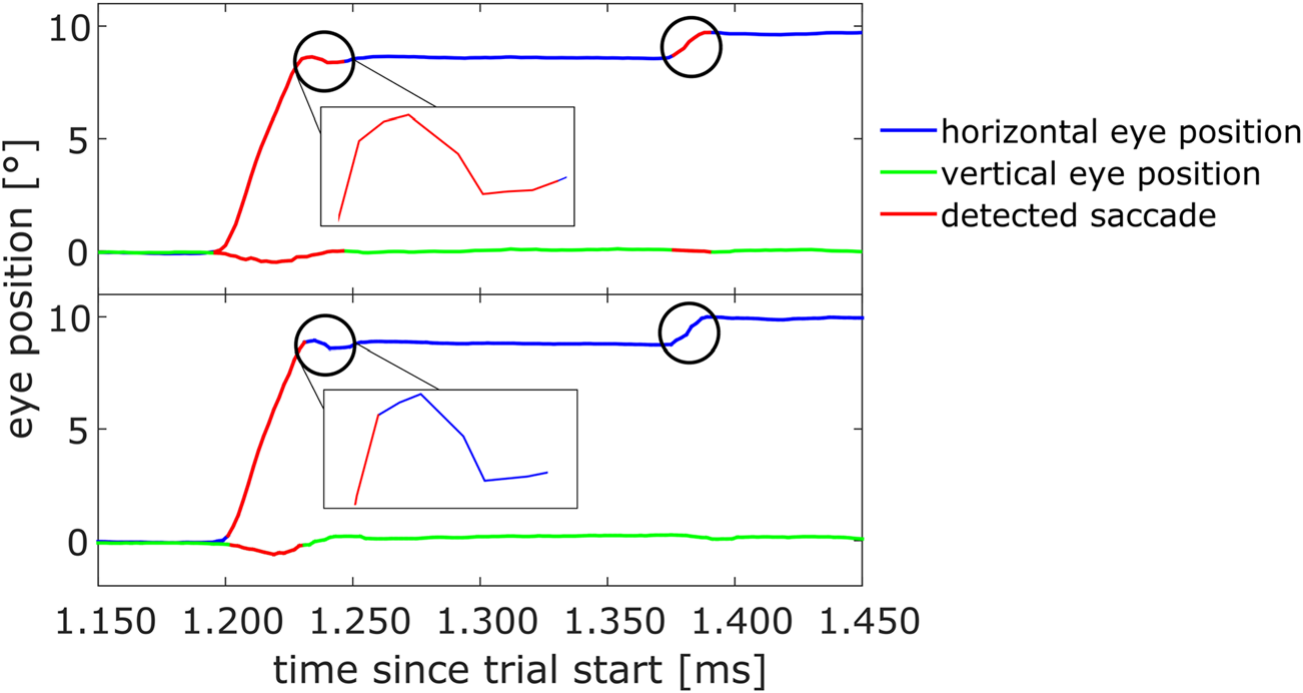
Illustration of the differences in saccade detection for the same raw eye position data between the standard EyeLink II (ELII; top) and the EyeSeeCam (ESC; bottom) saccade detector. The first encircled period in the top graph shows the last few data points associated to a saccade by the ELII detector. The same period was not assigned to the saccade by the ESC detector, since the position of the eye in that period did not change much, meaning that the velocity of the eye was below the threshold of 100°/s. This resulted in a shorter saccade duration and a slightly different saccade amplitude in the bottom graph. Furthermore, since only data points with a higher velocity were associated with the saccade by the ESC detector, the saccade mean velocity would most likely differ between the two types of saccade detector. The second encircled period marks a saccade with a small amplitude of about 1°. Here the ELII detector in the upper graph detects this eye movement as a saccade, whereas the ESC detector in the lower graph does not, because the eye velocity did not exceed the threshold for more than three consecutive samples

Indeed, the two standard saccade detection algorithms for the systems could produce slightly different results when used on the same eye trace (Fig. 2). In this example, two segments of the eye traces are of particular interest and are highlighted with circles. First, one can notice that the saccade detector used for the ESC (lower panel) attributes fewer data points to the first saccade than does the built-in saccade detector of the ELII (upper panel). This is due simply to the different velocity criteria (see above). This difference in thresholding leads to a considerably longer saccade duration for the ELII detector (50 ms, vs. 30 ms with the ESC detector), whereas the amplitudes of the saccade do not differ much (8.44° with the ELII vs. 8.63° with the ESC). Furthermore, a second, corrective saccade with an amplitude of about 1° is not detected by the ESC detector, since the velocity does not exceed the higher threshold in this case.

Finally, to accurately attribute potential differences to the specific hardware of the two eye-trackers, we also analyzed the raw eye-position data recorded by both systems with the same preprocessing algorithms—that is, the more conservative ESC saccade detector.

#### Fixation

In the first task, saccade and fixation periods alternated and allowed us to analyze the fixation performance. Here, a fixation period on a target began after the initial saccade (amplitude > 7°) toward that target, and ended with another saccade (amplitude > 7°) toward the next target. Consequently, small correction saccades and drifts that might occur during that fixation period were included into computation of the average absolute eye position. For a given trial, we analyzed the first fixation at the center of the screen, which served as a baseline reference, and the fixations at all eight eccentric target positions, as well as the second fixation of the central target. For a given subject, the absolute fixation error was computed separately for each stimulus position as the Euclidean distance between the target position and the averaged eye position during fixation of that target.

#### Smooth pursuit

In a last step, we computed the velocity gain during both smooth-pursuit tasks, linear and circular, to quantify target tracking. The gain is defined as the ratio of eye velocity and target velocity. This implies that an ideal smooth-pursuit eye movement, in which the eye velocity matches the target velocity, would have a gain of 1.0. If the gain is below one, the eye lags behind the pursuit target, whereas a value higher than one indicates that the eye is ahead of the target. To minimize saccadic distortions during smooth pursuit, we computed the eye velocity only during steady state pursuit phases, which begin about 300 ms after the initial saccade toward the pursuit target (Dowiasch, Blohm, & Bremmer, 2016b). Furthermore, data segments containing catch-up saccades during a pursuit phase, as detected by the respective saccade detector, were not used to compute the average velocity or gain from 40 ms before saccade onset until 60 ms after saccade offset.

#### Statistics

For our statistical analyses, we used a repeated measures analysis of variance (rANOVA). Repeated measures ANOVA is a commonly used statistical approach for multiple measures of the same variable taken on the same subjects under different conditions. In such a design, subjects serve as their own control. This reduces the influence of confounding covariates—for example, individual characteristics in the eye movements of subjects. Furthermore, they are statistically more efficient than a typical one-way ANOVA, and hence require fewer subjects (Jones & Kenward, 2014). Thus, the rANOVA is the equivalent of the one-way ANOVA, but for related, not independent, groups. As such, it can detect any overall differences between related means. It requires one independent categorical factor, also referred to as the *within-subjects factor*, and one dependent continuous variable. This means, in the present study, that the within-subjects factor is the type of eye-tracker used (ELII or ESC), and the dependent continuous variables are the different eye movement parameters, such as saccade amplitude.

In the following results, all data will be presented with their respective mean average across all eight subjects and the corresponding standard deviation (*SD*), as determined for each paradigm. Finally, we analyzed and compared the inter- and the intrasubject variability of all eye movement parameters. The *consistency or within-subjects variability* refers to the variability observed from repeated measurements on the same subject under the same experimental condition. On the contrary, the *intersubject or between-subject variability* is completely due to the heterogeneity between subjects and describes averaged differences in the eye movement parameters between all the subjects. In this study we chose to compare the inter-individual and intra-individual standard deviations as a measurement of variability. The interindividual standard deviations shown in Table 3 were calculated by first averaging across one eye movement condition (e.g., saccade direction) for each subject individually. Afterward, the standard deviation of these averages for each subject was calculated. Finally, the individual standard deviations for each subject were averaged across all subjects. Alternatively, the intra-individual standard deviation was calculated by first determining the standard deviation for each eye movement condition (e.g., saccade direction) for each subject individually. Afterward, the average of these standard deviations for each subject was calculated. Finally, the grand mean of these averaged standard deviations was calculated. This type of analysis allows us to determine whether one of the eye-trackers works well with some subjects but less well with others, which would be reflected in a higher intersubject variability, or whether the accuracy of an eye-tracker was generally worse for all subjects, which would be indicated by a higher intrasubject variability.

**Table 3:** Detailed list of all standard deviations (*SD*s) in the saccade parameters, both between subjects (interindividual) and within subjects (intra-individual)

Eye Movement Parameter	Interindividual *SD*		Intra-Individual *SD*	
	ELII	ESC	ELII	ESC
Saccade amplitude	0.40°	1.34°	0.89°	1.13°
Saccade mean velocity	18.27°/s	30.56°/s	33.82°/s	37.74°/s
Saccade peak velocity	35.37°/s	57.33°/s	55.44°/s	58.17°/s
Absolute fixation error	0.33°	1.20°	0.24°	0.31°
Smooth-pursuit gain	0.07	0.20	0.07	0.19

Although there were huge differences in the interindividual *SD*s between the two systems, these differences were markedly smaller for the intra-individual *SD*s

## Results

In this study we analyzed typical eye movement parameters recorded in prototypical oculomotor tasks. This allowed us to evaluate the fundamental performance properties of two video-based eye-tracking systems, the ESC and the ELII.

### Saccades and fixation

**Fig. 3:**
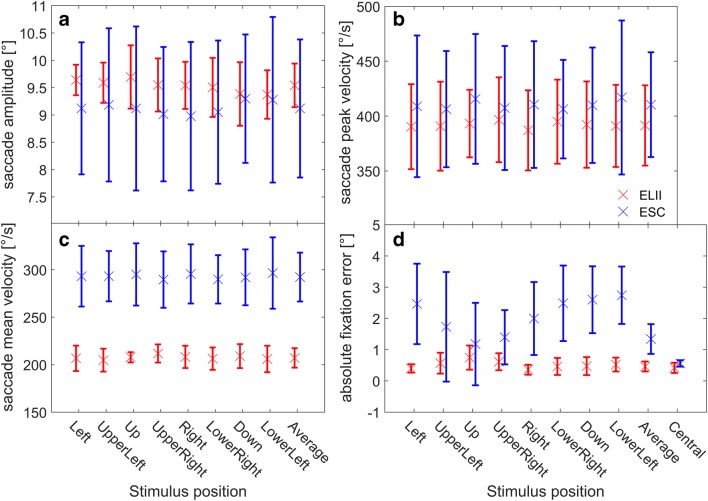
Population data for the four eye movement parameters in the saccade task, measured with the EyeLink II (ELII) and EyeSeeCam (ESC) and analyzed with their respective standard saccade detectors. The data and their standard deviations measured and analyzed with the ELII system and saccade detector are shown in red. The blue data points and standard deviations were measured and analyzed with the ESC system and saccade detector. (A) Saccade amplitudes for all eight stimulus positions with an eccentricity of 10°, and the average over all stimulus positions (mean ELII: 9.5 ± 0.4°; mean ESC: 9.1 ± 1.3°; *p* = .43). Both systems showed a characteristic saccadic undershoot for all directions. Noticeably, the standard deviation was much larger for the ESC data than for the ELII data. (B) Saccade peak velocities for all eight stimulus positions with an eccentricity of 10°, and the average over all stimulus positions (mean ELII: 391 ± 37°/s; mean ESC: 410 ± 48°/s; *p* = .40). (C) Saccade mean velocities for all eight stimulus positions with an eccentricity of 10°, and the average over all stimulus positions (mean ELII: 292 ± 26°/s; mean ESC: 207 ± 10°/s; *p* = 10^–4^). The saccade mean velocities measured and analyzed with the ESC were much higher for all eight stimulus positions and for the average, most likely because of the higher velocity threshold of the saccade detector used by this system. (D) Absolute fixation errors for all eight eccentric stimulus positions, the average over all stimulus positions (mean ELII: 0.5 ± 0.2°; mean ESC: 1.3 ± 0.5°; *p* = 4.14 × 10^–3^), and the absolute fixation errors for the central fixation spot. Here the ESC showed much larger errors than the ELII for all target positions except for the central fixation point. Furthermore, the magnitude of the error was modulated by the target position in both systems, but was more pronounced in the ESC

The analysis of the saccade task using each system’s default saccade detector showed no significant difference between the two eye-trackers concerning mean saccade amplitude to each of the eight peripheral targets or the average across all targets [mean ELII: 9.5 ± 0.4°, mean ESC: 9.1 ± 1.3°; *F*(1, 7) = 0.71, *p* = .43, rANOVA; Fig. 3A]. Furthermore, there was no significant difference between the two systems concerning mean saccade peak velocity [mean ELII: 391 ± 37°/s; mean ESC: 410 ± 48°/s; *F*(1, 7) = 0.80; *p* = .40, rANOVA; Fig. 3B]. In addition, we found no significant interaction between saccade direction and eye-tracking system for saccade amplitude [*F*(7, 49) = 1.76, *p* = .12, rANOVA] or saccade peak velocity [*F*(7, 49) = 0.61, *p* = .74, rANOVA]. As expected, both systems revealed small but consistent undershots for saccades in all directions. However, there was a significant difference between the two systems concerning the mean saccade velocities for all targets [mean ELII: 207 ± 10°/s; mean ESC: 292 ± 26°/s; *F*(1, 7) = 62.14, *p* = 10^–4^, rANOVA; Fig. 3C], but no interaction between saccade direction and the eye-tracker used [*F*(7, 49) = 0.92, *p* = .50, rANOVA]. Furthermore, we observed a significant difference between the ELII and the ESC concerning mean absolute fixation errors for the majority of the peripheral targets and for the average across all targets [mean ELII: 0.5 ± 0.2°; mean ESC: 1.3 ± 0.5°; *F*(1, 7) = 17.46, *p* = 4.14 × 10^–3^, rANOVA; Fig. 3D]. Finally, there was also a significant interaction between the location of the target and the eye-trackers [*F*(8, 56) = 7.22, *p* = 1.57 × 10^–6^, rANOVA]. The mean absolute fixation errors for both systems revealed a systematic pattern, although with a much smaller amplitude for the ELII than for the ESC. Although the errors of both systems for the central fixation point were rather small, the ESC showed much larger errors for targets in the lower than in the upper visual hemifield. In contrast, when using the ELII, the error was larger for targets in the upper visual hemifield than for targets in the lower visual hemifield.

**Fig. 4:**
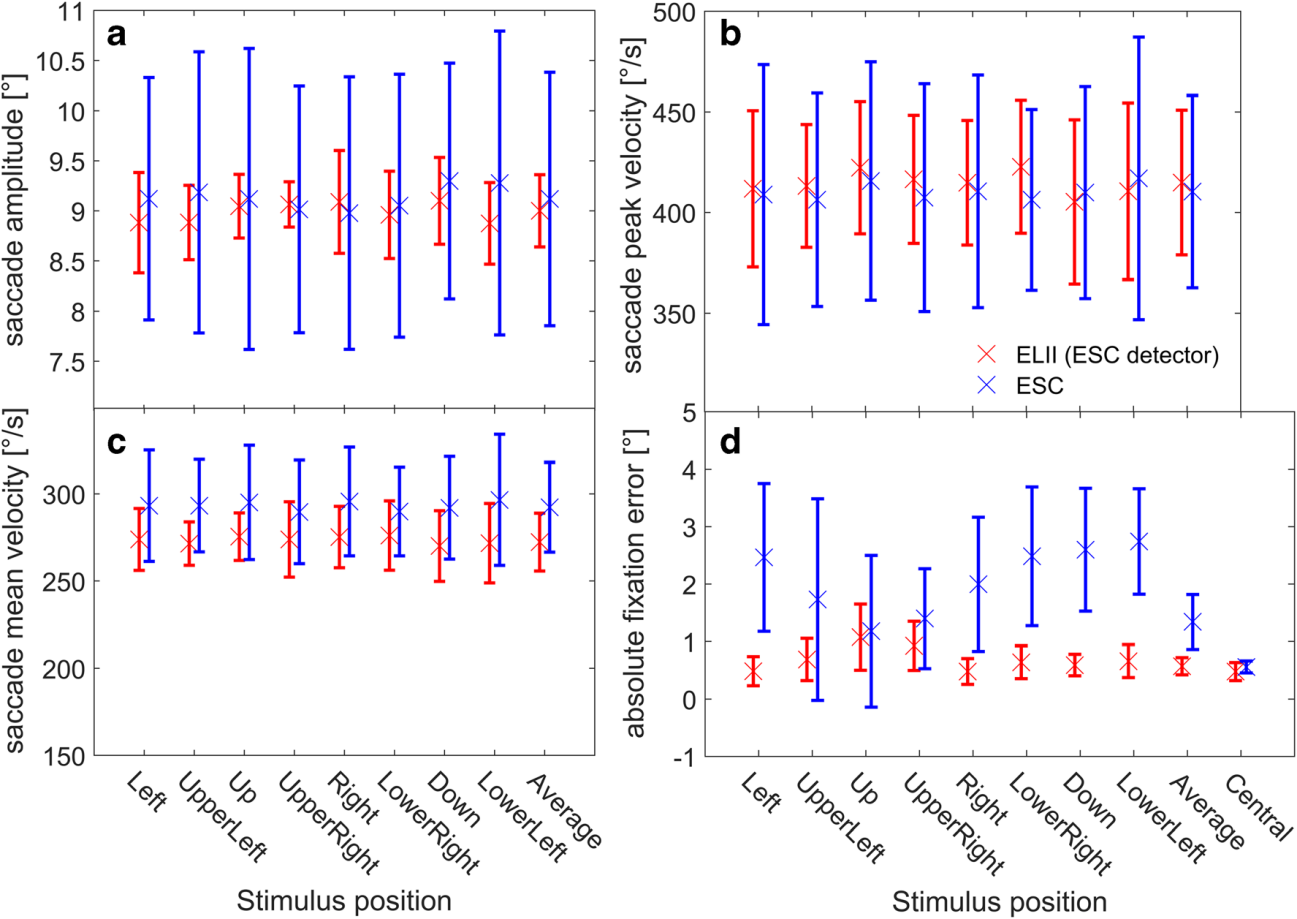
Population data for the four eye movement parameters in the saccade task measured with the EyeLink II (ELII) and EyeSeeCam (ESC) and analyzed with the same saccade detector (the ESC’s). The four subplots show the same parameters as in Fig. 3. This time, however, the same saccade detector was used to analyze both datasets. Data recorded with the ELII are shown in red, and data recorded with the ESC are in blue. (A, B) The different types of analysis did not change the overall results for saccade amplitude and peak velocity—that is, there was no significant difference between the two systems for these two parameters (mean amplitude ELII: 9.0 ± 0.4°; mean amplitude ESC: 9.1 ± 1.3; *p* = .78; mean peak velocity ELII: 415 ± 36°/s; mean peak velocity ESC: 410 ± 48°/s; *p* = .83). Moreover, the numerical differences got even smaller when both sets of raw data were analyzed by the same saccade detector. (C) Saccade mean velocities for all eight stimulus positions with an eccentricity of 10°, and the average over all stimulus positions. As before, both systems become much more similar when both were analyzed using the same saccade detector, resulting in no significant difference in the saccade mean velocities in any direction (mean ELII: 272 ± 17°/s; mean ESC: 292 ± 26°/s; *p* = .14). (D) As expected, the change in saccade detection did not change the results for absolute fixation errors, which were significantly higher for the ESC than for the ELII (mean ELII: 0.5 ± 0.1°; mean ESC: 1.3 ± 0.5°; *p* = 7.98 × 10^–3^)

To directly compare the raw data recorded by both eye-trackers, we performed a second analysis using the same saccade detector for the data from both systems. We decided on the saccade detector of the ESC for two reasons: First, it is more conservative than the detector of the ELII. Second, and most important, the source code of the ESC detector is openly available and can be directly applied to raw data from any eye-tracker. When using the same saccade detector, the saccade-related parameters for both systems were much more alike. Again, there was no significant difference between the two eye-trackers concerning mean saccade amplitude [mean ELII: 9.0 ± 0.4°; mean ESC: 9.1 ± 1.3°; *F*(1, 7) = 0.08, *p* = .78, rANOVA; Fig. 4A] or mean saccade peak velocity [mean ELII: 415 ± 36°/s; mean ESC: 410 ± 48°/s; *F*(1, 7) = 0.05, *p* = .83, rANOVA; Fig. 4B] for all peripheral targets and the average across all targets. Furthermore, with this analysis we found no significant difference between the two systems concerning the mean saccade velocities [ELII: 272 ± 17°/s; ESC: 292 ± 26°/s; *F*(1, 7) = 2.72, *p* = .14, rANOVA; Fig. 4C]. There was also no significant interaction between stimulus direction and the eye-tracker used for any of these parameters. Yet, because the influence of the saccade detection on the much longer fixations and their accuracy was low, there was still a significant difference between the two systems for the absolute fixation errors for most of the targets [ELII: 0.5 ± 0.1°; ESC: 1.3 ± 0.5°; *F*(1, 7) = 13.46, *p* = 7.98 × 10^–3^, rANOVA; Fig. 4D]. As in the previous analysis, we observed a systematic, direction-dependent modulation of the absolute fixation errors, resulting in a significant interaction between the two systems [*F*(8, 56) = 10.74, *p* = 5.22 × 10^–9^, rANOVA].

### Smooth pursuit

**Fig. 5:**
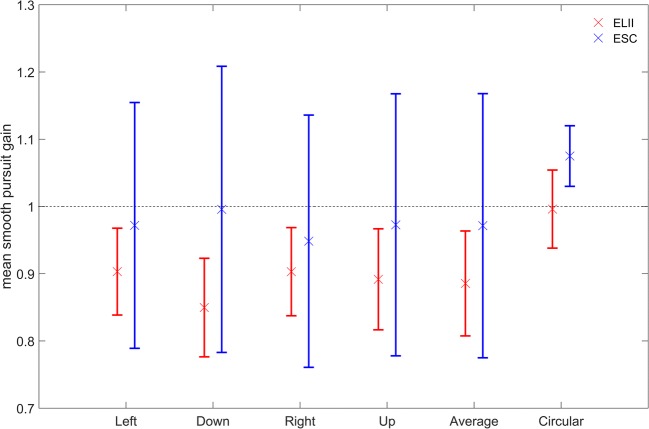
Population data of the smooth-pursuit gain in the step-ramp pursuit task and the circular pursuit task. The data and their standard deviations measured and analyzed with the EyeLink II (ELII) system and saccade detector are shown in red. Blue data points and standard deviations were measured and analyzed by the EyeSeeCam (ESC) system and saccade detector. Although the velocity gain of the step-ramp pursuit task was consistently higher for the ESC than for the ELII, the average gains did not differ significantly between the two systems (mean ELII: 0.88 ± 0.06°/s; mean ESC: 0.97 ± 0.19°/s; *p* = .28). Both systems showed a gain slightly below 1.0, resembling previous results in the literature. Yet there was a significant influence of pursuit direction on the gain in both systems (*p* = .01). This led to a larger difference between the systems for downward pursuit, whereas the difference was markedly smaller for rightward pursuit. Furthermore, the standard deviations of the gain during the step-ramp pursuit task were much larger for the ESC than for the ELII system. Finally, we observed a significant difference in velocity gain in the circular pursuit task (mean ELII: 1.00 ± 0.06°/s; mean ESC: 1.08 ± 0.05°/s; *p* = .02). In contrast to the step-ramp pursuit task, here the standard deviations of both systems were almost equal

For the linear smooth-pursuit eye movement task, both systems showed mean velocity gain values slightly below the ideal value of 1.0, which is in line with the literature, and the values were not significantly different [mean ELII: 0.88 ± 0.06°/s; mean ESC: 0.97 ± 0.19°/s; *F*(1, 7) = 1.34, *p* = .28, rANOVA; Fig. 5]. Yet there was a significant interaction between pursuit direction and the eye-tracking system used [*F*(3, 21) = 4.41, *p* = .01, rANOVA]: The difference in velocity gain between the two eye-tracking systems was largest for downward pursuit and smallest for rightward pursuit. On the other hand, the mean velocity gains during circular pursuit were slightly higher for both systems than during linear pursuit [mean ELII: 1.00 ± 0.06°/s; mean ESC: 1.08 ± 0.05°/s; Fig. 5]. Unlike with linear pursuit, here the difference between the two eye-trackers was statistically significant (*t* = – 3.14, *p* = .02, *t* test).

### General aspects

**Fig. 6:**
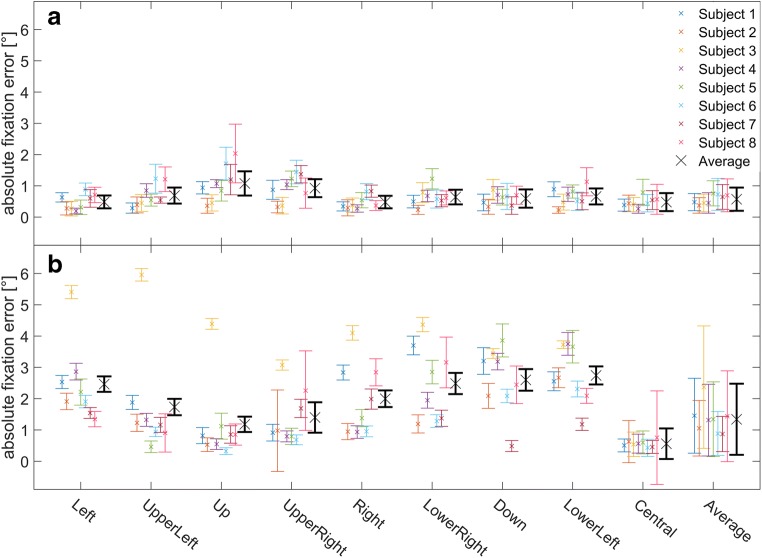
Data for the absolute fixation errors in the saccade task on a single-subject level. Here the absolute fixation errors of all individual subjects are shown color-coded for all eight eccentric target locations, the central fixation target, and the averaged across all stimulus positions. Furthermore, the average over all subjects for each stimulus position is shown in black. The upper graph shows the EyeLink II (ELII) data analyzed with the EyeSeeCam (ESC) saccade detector, and the lower graph shows the ESC data analyzed with the ESC saccade detector. The ELII system showed much higher uniformity across subjects and stimulus locations than did the ESC. Furthermore, this analysis revealed one subject (Subject 3) with a huge position-dependent fixation error when using the ESC, whereas there was no such error with the ELII system. There was no noticeable difference between the two systems for the central fixation target on a single-subject level

Noticeably, for almost all of the investigated parameters, the standard deviation was higher for measurements performed with the ESC than for those with the ELII. To analyze this effect and its source in more detail, we computed the interindividual (between-subjects) and intra-individual (between individual trials within each subject) errors for all previously presented eye movement parameters. The results are depicted in Table 3. The data show that for all eye movement parameters, the intra-individual *SD*s were at almost the same level for both systems, whereas the interindividual *SD*s were much larger for the ESC than for the ELII. In Fig. 6, the absolute fixation error for each subject and each target for both eye-trackers is shown, as a representative example. The ELII system (Fig. 6A) provided more constant fixation data across subjects and stimulus locations than did the ESC (Fig. 6B). Furthermore, one subject (Subject 3) had huge position-dependent absolute fixation errors only when using the ESC. This might have been due to the fact that Subject 3 was the only participant wearing glasses, which did not cause any problems when using the ELII but did interfere with the ESC. Yet the other subjects also showed higher interindividual *SD*s when using the ESC. In addition, direction-dependent modulation of the absolute fixation errors was also evident at the single-subject level for both systems.

## Discussion

In this study we compared the performance of two video-based eye-tracking systems, the fully mobile EyeSeeCam and the head-mounted but laboratory-bound EyeLink II, during typical oculomotor experiments. While the ELII can be considered a gold standard in oculomotor research in the laboratory, no comparable quantitative data from the laboratory are available for the ESC. However, the latter device has already been used with great success in a number of real-world eye movement studies (Dowiasch, Backasch, et al., 2016a; Dowiasch et al., 2015; Korsager et al., 2016; Kugler et al., 2015; Marx et al., 2012; B. M. ’t Hart & Einhäuser, 2012). The accuracy of the ESC and other mobile eye-trackers in these settings indicate slightly worse accuracy in natural environments than in tests performed in the laboratory, as reported by these and other studies (MacInnes et al., 2018). However, here we could show that, despite their different hardware features, their different methods for computing gaze direction and, more general, their different fields of key application, the ESC and the ELII provided indistinguishable data at the population level in almost all cases. This result is of critical importance since current and future eye movement studies will focus more and more on recordings in natural settings—that is, outside the laboratory and without any restraint of the head or body. Our data also showed that a standardized analysis of the raw data with identical parameters across different eye-trackers—for example, for saccade detection—is often more important for consistent results across systems, than the hardware itself. Even two completely different gaze estimation techniques—that is, a 3-D model of the eyeball for the ESC and a vector between the center of the dark pupil and the corneal reflection of the infrared illumination unit for the ELII—provide similar data on the population level if the raw data are analyzed by the same detector.

### Saccades

First, we analyzed typical saccade parameters like amplitude and velocity, in a task in which subjects had to perform a visually guided saccade with an amplitude of 10° to one out of eight predefined centrifugal targets. We could show that the main difference between the data from the two eye-trackers was the computed mean saccade velocity. Yet, this difference emerged due to the different standard saccade detectors employed by both systems to analyze the raw data. These two detectors are a consequence of the different fields of application both eye-trackers are typically used in. The ELII is used in the laboratory with constant infrared illumination of the eye. This setting minimizes the occurrence of potential artifacts and allows a more liberal velocity threshold in order to maximize the yield. On the other hand, in natural environments and during head and self-motion the ESC is more likely to face interfering eye movements like VOR or light reflexes from external illumination (Dowiasch, Backasch, et al., 2016a; Dowiasch et al., 2015). To exclude these intrusions from a robust saccade-based analysis, the detection threshold has to be set to a much higher velocity value. This conservative approach guarantees a low false detection rate of saccades in a natural environment, while sacrificing the detection of small saccades with an amplitude of about 1° or less. Furthermore, a higher velocity threshold might omit a few data points at the beginning or end of a saccade, resulting in less low-velocity data points belonging to a saccade. Since a typical 10° saccade usually only takes about 40 ms (Bahill, Stark, & Clark, 1975), which corresponds to roughly 12 data samples of the ESC and 20 data sample from the ELII, the two different saccade detection algorithms (ESC vs. ELII) can increase this discrepancy even further and therefore obviously do have a huge impact on resulting mean saccade velocity. Furthermore, the different thresholds of both saccade detectors lead to a slight difference between the timing of start and end point when used on the same saccade. This results in a different saccade duration computed by both algorithms and a slightly smaller saccade amplitude for higher velocity thresholds. Accordingly, when the raw data of both eye-trackers were analyzed with the same saccade detector, there was no longer a significant difference between the two systems for any saccade-related parameter. This suggests that the ESC is capable of providing saccade parameters with a sufficient precision for oculomotor research. This seems to be the case despite the hardware differences as compared to a standard laboratory eye-tracker—for example, resulting in a lower sampling frequency. Both systems, ESC and ELII, provided saccade mean and peak velocities in the same range as predicted by the saccadic main sequence for saccades with an amplitude of 10°. Furthermore, the characteristic saccadic undershoot of roughly 10% of the saccade amplitude (van der Heijden, van der Geest, De Leeuw, Krikke, & Müsseler, 1999), meaning that saccade amplitudes are often a bit smaller than the actual distance between the targets, could be reliably reproduced in our study with both eye-tracking systems.

### Fixation

In a second step, we analyzed and compared the fixation accuracy of both eye-tracking systems at the central fixation target and the eight peripheral targets used in the saccade task. Here, our study revealed a significantly larger absolute fixation error for the ESC than for the ELII. Yet both eye-trackers showed average fixations errors similar to those of other stationary (around 0.5°) or mobile (around 1.5°; Blake, 2013) eye-trackers. We couldn’t find any significant difference between both systems concerning the relative fixation error. This suggests that in general the ESC is capable of providing data with the same precision as the ELII, but it has a worse overall accuracy. This might be due to the fact that the calibration procedures differ between the two systems. First of all, the ELII uses nine predefined targets to match the gaze of a subject to their calibration targets on the screen—that is, one central point, and other points at the top, bottom, left, and right sides and the four corners of the screen. The latter four targets were not used by the standard ESC calibration procedure, which only uses five calibration targets. Previous studies have shown that a larger number of calibration targets usually increases gaze accuracy especially for peripheral targets (Duchowski, 2007; SR Research Ltd, 2006). These previous findings are supported by our present data. Here the mean absolute fixation errors for the central target were 0.56° for the ESC and 0.42° for the ELII, which were not statistically different (*p* > .05, *t* test). Yet, for peripheral targets, the error increased markedly for the ESC (between 1.18° and 2.74°), whereas it stayed almost constant for the ELII system (between 0.36° and 0.74°).

Furthermore, not all subjects could be calibrated optimally with the ESC, because glasses produced ambiguous interferences for pupil detection. This was especially true for Subject 3, who showed the highest fixation errors. On the other hand, the ELII did not seem to have a problem with the glasses of this subject, since the fixation errors measured with the ELII were within the typical range of the other subjects. This might be the case because the eye cameras of the ELII system are moveable and can be adjusted to a wide range of different positions relative to the eye to avoid positions with inferior pupil detection quality or artifacts. The ESC on the other hand, only offers a fine adjustment of the cameras to bring the pupil in the center of the camera view, while the angle of the camera relative to the eye is fixed. Yet the calibration procedure of the ESC does have one major advantage as compared to the ELII: The ESC uses a sophisticated eyeball model in order to be able to operate even without any calibration if needed (Stoll, 2015). The parameters of the model were usually measured before the first recording (e.g., eye distance) or were automatically adjusted to each subject during the calibration procedure. The latter might work well for some subjects, and worse for others (e.g., if the subject has a slight strabismus). On the other hand, this allows measuring almost every subject with at least decent accuracy, whereas other eye-trackers would not be able to record reliable data without proper calibration. Especially in clinical trials and for subjects with rare diseases, in which complex calibration procedures might sometimes be too demanding, this is a huge advantage of the ESC (Marx et al., 2012). Finally, the fixations of the different targets in the saccade paradigm revealed a significant interaction between both eye-trackers and the stimulus position, resulting in a systematic pattern of the absolute fixation error. The amplitude of this pattern was much larger for the ESC than for the ELII and revealed a phase shift between both systems by roughly 180°. Whereas for the ESC the errors across subjects were smallest in the upper hemifield and largest in the lower hemifield, the situation was opposite for the ELII. This effect was present even at a single-subject level. This error pattern might be a direct result of the different relative arrangements of the components of the eye-trackers and especially the different camera positions relative to the eyes as used by both systems. The ELII uses cameras that record a video image of the eye directly from below. Hence, when a subject looks upward the pupil is distorted to an ellipse, for which the software might only partially be able to compensate. On the other hand, the ESC uses hot mirrors to reflect the infrared light to cameras above the eyes. These hot mirrors are placed almost directly in front of the eyes, which minimizes the elliptical distortion of the pupil for eccentric positions and allows a more compact construction of the eye-tracker. Yet, from this camera angle, eyelashes might produce more artifacts especially when looking downward.

### Smooth pursuit

In a second paradigm, we analyzed eye movement gain during a linear step-ramp pursuit eye movement in the four cardinal directions with both eye-trackers. Here, the results did not show any significant differences between the performances of both eye-trackers. Furthermore, the results of both eye-trackers and all directions were in line with previous studies, which showed that for pursuit with the speed of 10°/s the eyes usually slightly lag behind the pursuit target (Dowiasch, Blohm, & Bremmer, 2016b; Schlack, Hoffmann, & Bremmer, 2003), resulting in a velocity gain slightly below 1.0—that is, between 0.95 and 0.98 (Rottach et al., 1996). In the third paradigm—that is, investigating circular smooth pursuit—we found a statistical difference between the performance of the two eye-trackers concerning the mean velocity gain. Interestingly, here the gain was higher than during linear pursuit for both systems, with values above 1.0, suggesting that the eyes moved faster than the target. This is in line with previous results (Rottach et al., 1996) and might just be an artifact of the Euclidian combination of the horizontal and vertical velocity components during this type of eye movement. Analyzed separately, both directional components in our study were well below 1.0. The reason for the significant difference in this paradigm as compared to linear pursuit could be that the circular pursuit was longer than the linear pursuit (3.9 s vs. 2 s) and has been described as more challenging (De Brouwer, Yuksel, Blohm, Missal, & Lefevre, 2002). This might have led to an increased number of catch-up saccades during circular pursuit. Since the ESC and its more conservative saccade velocity threshold detected small saccades less reliably, more eye movement segments with high eye velocities might have been used for computing the gain of the ESC measurement as compared to the EL II.

Finally, the standard deviations of the eye movement data recorded with the ESC were larger than those measured for the EL II for almost all parameters investigated. Although the intra-individual variance of the ESC data implies, that the ESC is generally capable to perform measurements as precise as the ELII, the higher interindividual variance of the ESC data suggests that the performance depends highly on the calibration of the eye-tracker for a given subject. For instance, one of the subjects in our study could not be calibrated with sufficient accuracy at the peripheral targets due to interferences with the subjects’ glasses. In general, there was a higher variance of eye movement parameters between subjects with good calibration and those with poorer calibration for the ESC, whereas all subjects could be calibrated equally well using the ELII. Yet, even with a poor or no calibration at all, the ESC is able to provide relative eye movement parameters, like eye velocities and amplitudes, with reasonable quality (Stoll, 2015). This unique feature has been proven to be particularly important in clinical studies in which a proper calibration sometimes is impossible due to disease related dysfunctions (Marx et al., 2012).

In conclusion, although designed as a fully mobile, lightweight eye-tracker for natural environments, the ESC provides quantitative eye movement parameters that are fully suited for typical psychophysical and oculomotor studies. Only studies investigating very specific eye movement parameters—for example, microsaccades—or that heavily focus on absolute values, such as for saccade duration, would require a more sophisticated eye-tracker, which then, however, would have to be used in the laboratory. Moreover, our study revealed that the different standard analysis procedures of different eye-trackers (e.g., built-in saccade detectors) caused larger deviation of the results than the hardware used to record the raw data. This important factor has to be taken into account when comparing results of different studies carried out with different eye-trackers in different environments. Since recently more and more eye movement studies were carried out in natural environments to reveal a deeper insight and provide a more complete understanding of the visual system, it would be desirable to establish a guideline regarding comparability and reproducibility with commonly known laboratory results. Our present study takes a first step toward this merging of knowledge from eye-tracking in the well-controlled, high-performance but artificial laboratory and the widely uncharted by more natural real-world eye-tracking.

#### Author note

This research was supported by Deutsche Forschungsgemeinschaft Grants IRTG-1901 and CRC/TRR-135 (Project number 222641018) and by the German Federal Ministry of Education and Research (BMBF), Project DIADEM. The study was planned by S.D. and F.B. Bachelor’s student P.W. conducted the experiment after instruction and training by S.D. S.D. and P.W. did the analyses of the experiments. Part of the data were included in the bachelor’s thesis of P.W., conducted under the supervision of F.B. The manuscript was written by S.D., P.W., and F.B. The authors declare no conflict of interests. Datasets are available at 10.5281/zenodo.1434736
